# Circulating MiRNA-195-5p and -451a in Diabetic Patients with Transient and Acute Ischemic Stroke in the Emergency Department

**DOI:** 10.3390/ijms21207615

**Published:** 2020-10-15

**Authors:** Mauro Giordano, Maria Consiglia Trotta, Tiziana Ciarambino, Michele D’Amico, Marilena Galdiero, Federico Schettini, Diego Paternosto, Marta Salzillo, Roberto Alfano, Vincenzo Andreone, Lorenzo Salvatore Malatino, Gianni Biolo, Giuseppe Paolisso, Luigi Elio Adinolfi

**Affiliations:** 1 Department of Advanced Medical and Surgical Sciences, University of Campania “L. Vanvitelli”, 80138 Naples, Italy; mauro.giordano@unicampania.it (M.G.); fede.skett@gmail.com (F.S.); giuseppe.paolisso@unicampania.it (G.P.); luigielio.adinolfi@unicampania.it (L.E.A.); 2 Department of Experimental Medicine, Division of Pharmacology, University of Campania “L. Vanvitelli”, 80138 Naples, Italy; mariaconsiglia.trotta2@unicampania.it (M.C.T.); marilena.galdiero@unicampania.it (M.G.); roberto.alfano@unicampania.it (R.A.); 3 Department of Internal Medicine, Hospital of Marcianise, ASL, 81025 Caserta, Italy; tiziana.ciarambino@gmail.com; 4 Department of Emergency Medicine, AORN Sant’Anna e San Sebastiano,81100 Caserta, Italy; paternosto58@gmail.com (D.P.); martasalzillo@tin.it (M.S.); andreone2@gmail.com (V.A.); 5 Department of Clinical and Experimental Medicine, University of Catania, 95126 Catania, Italy; malatino@unict.it; 6 Department of Medical and Surgical Sciences, University of Trieste, 34100 Trieste, Italy; biolo@units.it

**Keywords:** microRNA, acute ischemic stroke, transient ischemic attack, diabetes mellitus, emergency

## Abstract

(1) Background: Circulating micro-RNAs (miRNAs) modulate the expression of molecules in diabetes. We evaluated the expression of serum miRNA-195-5p and -451a in diabetic patients with ischemic stroke and correlated them with two markers of brain tissue integrity. (2) Methods: Seventy-eight subjects with acute ischemic stroke (AIS) or transient ischemic attack (TIA) (40 with diabetes) were enrolled. Serum miRNA levels, brain-derived neurotrophic factor (BDNF) and vascular endothelial growth factor A (VEGF-A) were assessed at admission and 24 and 72 h after a post-ischemic stroke, and were compared to 20 controls. (3) Results: Both circulating miRNAs were two-fold up-regulated in diabetic AIS and TIA patients compared to non-diabetics. Their levels progressively decreased at 24 and 72 h in both AIS and TIA patients. Interestingly, in the non-diabetic TIA group, both circulating miRNAs, although higher than the controls, tended to achieve a complete decay after 72 h. Furthermore, miRNA-195-5p and miRNA-451a levels inversely correlated with both BDNF and VEGF-A serum levels. (4) Conclusions: These data show a different profile of both micro-RNAs in diabetic versus non-diabetic patients after acute ischemic stroke, suggesting their pivotal role in cerebrovascular ischemic attack.

## 1. Introduction

Previous data have reported the involvement of several micro-RNAs (miRNAs) in post-ischemic angiogenesis as a crucial step to restore blood supply to ischemic cerebral regions after stroke [1,2].

Among them, hypoxia-regulated miRNAs (HRMs) play a crucial role, since they are highly expressed following cerebral hypoxic conditions, due to cerebral blood flow reduction [3].

In particular, cerebral HRMs, such as miRNA-195-5p and miRNA-451a, have been both reported to suppress vascular endothelial growth factor A (VEGF-A) levels, thereby reducing new vessel formation in hepatocellular carcinoma in a rat brain after stroke [4,5,6]. We previously reported an increased expression of circulating miRNA-195-5p and miRNA-451a levels in patients with both acute ischemic stroke (AIS) and transient ischemic attack (TIA) at admission [6]. However, after 48 h of the post-ischemic period, we found reduced miRNA serum levels according to the ischemic event severity, so it is unclear if miRNA-195-5p and miRNA-451a circulating levels show a decay over time. Moreover, so far, no relationship of between either miRNA-195-5p or miRNA-451a and the mechanisms of angiogenesis after ischemic stroke has been reported in patients. As a matter of fact, angiogenesis is crucial not only to restore blood flow to the ischemic cerebral area, but also to promote neurogenesis and to improve neurological functions [7]. Thus, miRNA-195-5p and miRNA-451a could be involved in the mechanisms that restore blood supply to the ischemic area after AIS. Moreover, a possible correlation between miRNA-195-5p and miRNA-451a expression with both BDNF and VEGF-A serum levels has never been reported. In contrast, diabetes mellitus is a recognized independent risk factor of stroke and is associated with higher mortality [8,9], particularly the complex interplay of several inflammatory and metabolic aspects in diabetic patients strongly affecting the cardio-vascular system [10,11]. Moreover, a frequent association of cerebrovascular events (AIS or TIA) with diabetic feet has been previously reported [10]. However, to the best of our knowledge, no data have been reported on the possible changes in circulating miRNA-195-5p and miRNA-451a levels in diabetic patients with AIS.

Therefore, in the present study, we performed an evaluation of circulating miRNA-195-5p and miRNA-451a profiles in diabetic and non-diabetic subjects with AIS and TIA, at 24 and 72 h during the post-ischemic period. We also evaluated a possible correlation between miRNA-195-5p and miRNA-451 expression with both BDNF and VEGF-A serum levels.

## 2. Results

### 2.1. Characteristics of the Study Subjects

The clinical characteristics in the control group and in the diabetic (D) and non-diabetic (ND) patients with AIS and TIA are reported in Table 1. In particular, there were no significant differences in the number or gender of patients among the control subjects, or the diabetic and non-diabetic stroke populations. Both diabetic AIS and TIA patients showed similarly long durations of diabetes, as well as similar glycated hemoglobin values. Moreover, body mass index, systolic and diastolic blood pressure, hypertension, smoking, and hyperlipidemia did not differ significantly among groups.

### 2.2. Main Results

#### 2.2.1. miRNA-195-5p and miRNA-451a in Non-Diabetic and Diabetic Patients with AIS

**Figure 1:**
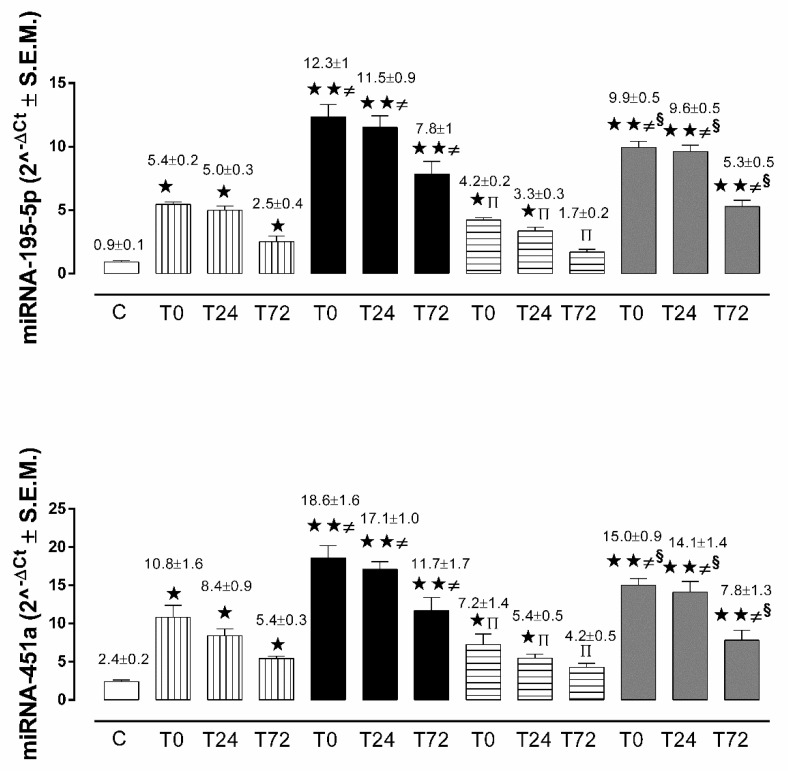
Micro-RNA (miRNA)-195-5p and miRNA-451a expression levels as mean of 2^^-ΔCt^ ± S.E.M. in control subjects (white column), in non-diabetic AIS patients (vertical line column), in non-diabetic TIA patients (horizontal line column), in diabetic AIS patients (black column), and in diabetic TIA patients (grey column). 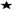 *p* < 0.05 vs. C; 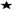

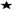
 *p* < 0.01 vs. C; **≠** *p* < 0.05 vs. ND at the same time point; ^π^ *p* < 0.05 vs. non-diabetic AIS at same time point; **^§^** *p* < 0.05 vs. diabetic AIS at same time point.

In the non-diabetic patients with AIS, circulating miRNA-195-5p and miRNA-451a levels were significantly up-regulated at admission (T0) and at 24 h (T24) and 72 h (T72) during the post-ischemic period in comparison to the control group (Figure 1).

At all time points, circulating miRNA-195-5p and miRNA-451a levels were significantly more up-regulated in diabetic patients with AIS in comparison to both the control and non-diabetic AIS groups (Figure 1).

#### 2.2.2. miRNA-195-5p and miRNA-451a in Non-Diabetic and Diabetic Patients with TIA

In the non-diabetic patients with TIA, circulating miRNA-195-5p and miRNA-451a levels were significantly up-regulated at admission (T0) in comparison to the controls, while there were no statistically significant changes after 72 h (T72) (Figure 1). At T0, T24, and T72, circulating miRNA-195-5p and miRNA-451a levels were significantly more up-regulated in diabetic patients with TIA, in comparison to both the control and non-diabetic TIA groups (Figure 1). Moreover, TIA patients showed significantly down-regulated miRNA-195-5p and miRNA-451a levels, as compared to AIS patients, in both non-diabetic and diabetic subsets (Figure 1).

#### 2.2.3. Brain-Derived Neurotrophic Factor (BDNF) and Vascular Endothelial Grow Factor A (VEGF-A) in Non-Diabetic and Diabetic Patients with AIS

**Figure 2:**
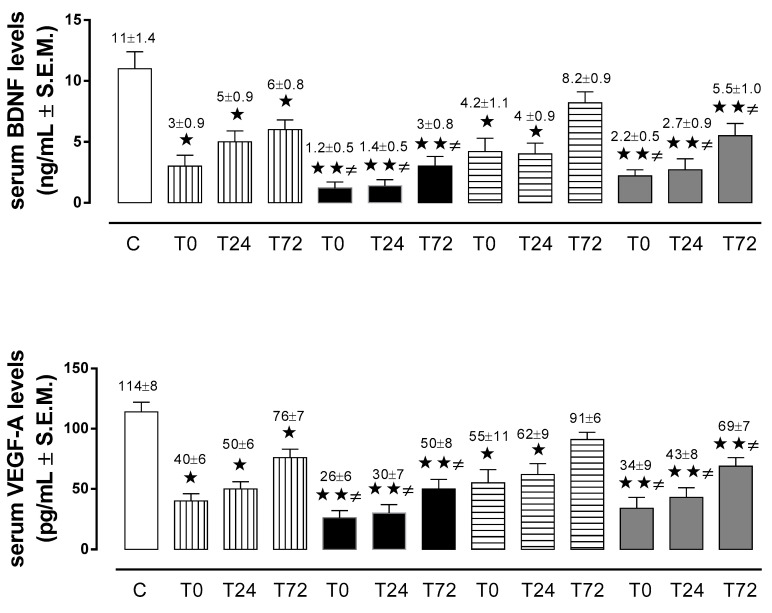
Serum levels of brain-derived neurotrophic factor (BDNF; ng/mL ± S.E.M.) and vascular endothelial growth factor A (VEGF-A; pg/mL ± S.E.M.) in control subjects (white column), in non-diabetic AIS patients (vertical line column), in non-diabetic TIA patients (horizontal line column), in diabetic AIS patients (black column), and in diabetic TIA patients (grey column). 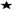 *p* < 0.05 vs. C; 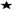

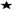
 *p* < 0.01 vs. C; **≠** *p* < 0.05 vs. ND at same time point.

In the non-diabetic patients with AIS, BDNF and VEGF-A serum levels were significantly lower than in the control group at admission (T0), at 24 h (T24), and 72 h (T72) during the post-ischemic period. Similarly, in the diabetic AIS group, BDNF and VEGF-A serum levels were significantly more reduced at all time points, in comparison with both controls and non-diabetic patients with AIS (Figure 2).

#### 2.2.4. BDNF and VEGF-A in Non-Diabetic and Diabetic Patients with TIA

In the non-diabetic patients with TIA, BDNF and VEGF-A serum levels were significantly lower than in the control group at admission (T0) and at 24 h (T24), while there were no statistically significant changes at 72 h (T72) during the post-ischemic period. In contrast, in the diabetic patients with TIA, BDNF and VEGF-A serum levels were significantly lower at T0, T24, and T72, in comparison to both the control and non-diabetic TIA groups (Figure 2).

#### 2.2.5. Correlation between miRNAs and BDNF or VEGF-A Serum Levels

**Figure 3:**
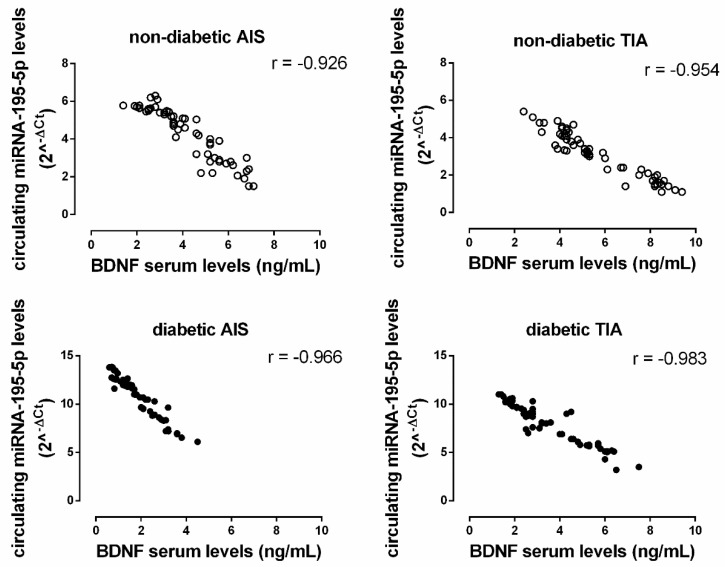
The correlation between miRNA-195-5p expression levels (2^^-ΔCt^; *y*-axis) with the serum levels of brain-derived neurotrophic factor (BDNF; ng/mL; *x*-axis) in both non-diabetic (ND; empty circle) and diabetic (D; solid black circle) AIS and TIA groups. All correlations are significant at *p* < 0.05.

In the non-diabetic patient population, a significant negative correlation was observed between miRNA 195-5p levels (2^^-ΔCt^) and BDNF serum levels (AIS: r = −0.926, *p* < 0.05; TIA: r = −0.953, *p* < 0.05). This negative correlation was also observed in the diabetic population (AIS: r = −0.966, *p* < 0.05; TIA: r = −0.983, *p* < 0.05) (Figure 3).

**Figure 4:**
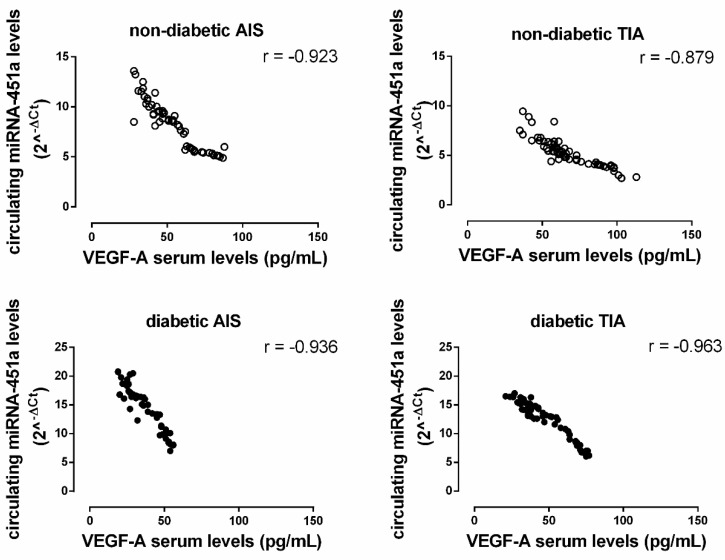
The correlation between miRNA-451a levels (2^^-ΔCt^; *y*-axis) with the serum levels of vascular endothelial growth factor-alpha (VEGF-A; pg/mL; *x*-axis) in both non-diabetic (ND; empty circle) and diabetic (D; solid black circle) AIS and TIA groups. All correlations are significant at *p* < 0.05.

A significant negative correlation between miRNA-451a levels (2^^-ΔCt^) and VEGF-A serum levels was observed in the non-diabetic population (AIS: r = −0.923, *p* < 0.05; TIA: r = −0.879, *p* < 0.05). The same negative correlation was observed in the diabetic population (AIS: r = −0.936, *p* < 0.05; TIA: r = −0.963, *p* < 0.05) (Figure 4).

## 3. Discussion

Our results demonstrate, for the first time, that diabetic patients with either AIS or TIA are associated with a marked increase in miRNA-195-5p and miRNA-451a profiles. We previously reported, in non-diabetic patients with either AIS or TIA, a higher expression of miRNA-195-5p and miRNA-451a [6]. To this regard, our data demonstrate that, in comparison with TIA, AIS patients always show a higher expression of miRNA-195-5p and miRNA-451a, whether diabetic or non-diabetic. Although it is already known that ischemic stroke subtypes are associated with different prognoses and that AIS patients show a specific HLA and killer cell immunoglobulin-like receptor (KIR) genotyping [12,13], this different expression of both miRNAs may suggest their role as biomarkers of brain tissue damage severity.

In the present study, we also observed, in non-diabetic patients with TIA, a complete decay of both circulating miRNAs 72 h after an ischemic stroke. On the contrary, this profile of miRNAs was not observed in the diabetic group, suggesting that the more pronounced miRNA expression in diabetic subjects needs a longer period of time for complete decay. We do not know the exact mechanism of such a finding. However, it cannot be excluded that the blunted decay could be due to the major number of copies of miRNAs released into circulation within 72 h. In this regard, previous studies have reported a trend of decay over time of miRNAs after traumatic injury [14]. However, the behavior of circulating miRNA-195-5p and miRNA-451a levels in patients with AIS or TIA 72 h after an ischemic stroke has not previously been evaluated. In particular, we observed, in non-diabetic patients with TIA, that the decay of miRNA-195-5p 72 h after the ischemic stroke period was associated with a marked increase in BDNF serum levels. This pattern of BDNF might indicate an attempt to induce neuronal recovery from the brain damage that occurs in the post-ischemic stroke period. By contrast, in diabetic patients with either TIA or AIS, the increase in BDNF after 72 h did not achieve non-diabetic levels. Significantly, BDNF levels in diabetic patients were already much lower at baseline (T0). This BDNF pattern may suggest that, after an ischemic event, diabetic subjects are not only characterized by more severe brain damage in comparison to non-diabetic patients, but are also already less protected at baseline.

In the present study, we also observed an inverse relationship between BDNF and the miRNA-195-5p profile in the post-ischemic stroke period, suggesting that peaks of miRNA-195-5p and BDNF are asynchronous, thereby representing two different steps in post-ischemic neuronal repair.

We also reported that, in diabetic patients with TIA, an increased expression in miRNA-451a was associated with lower levels of circulating VEGF-A and this was even more markedly reduced in diabetic patients with AIS. This may suggest larger vascular endothelial damage in diabetic patients with AIS. However, we noted that diabetic patients started with lower VEGF-A levels at baseline, and this probably explains the lower levels even after 72 h. In contrast, in non-diabetic patients with TIA, 72 h after a post-ischemic event, VEGF-A levels tended to return to the levels of the control group, suggesting a potential improvement of the vascular milieu in the recovery phase after an ischemic event.

These data are also in agreement with previous evidence of VEGF-A on neurogenesis and improvement of neurological functions [15,16]. In this regard, in the present study, we also reported for the first time a correlation between miRNA-451-a expression and VEGF-A serum levels in the post-ischemic period.

The main limitations of this study are represented by the real-time reverse transcription (qRT-PCR) procedures. At the moment, this assay requires several hours to be processed, but it cannot be excluded that in the future this technique could hopefully be analyzed faster and be more useful in an emergency clinical setting. In addition, our study enrolled a small number of patients in a single center. Unfortunately, we did not evaluate the severity of stroke, as it was not in the main aim of the study. However, this is a “proof of concept” study, opening the door to further larger studies aimed at evaluating a possible correlation between the severity of strokes with miRNAs and/or VEGF-A/BDNF. This will better clarify the potential involvement of miRNA expression in the pathogenetic chain of ischemic stroke and its severity.

## 4. Materials and Methods

This study was performed at the Hospital of Marcianise, ASL Caserta, Italy. We evaluated the circulating miRNA-195-5p and miRNA-451a profiles in diabetic and non-diabetic subjects with AIS and TIA at 24 and 72 h during the post-ischemic period. The study population included 98 subjects: 20 patients without a history of ischemic stroke (control group (C)), 37 patients with TIA (19 with diabetes), and 41 patients with AIS (21 with diabetes). The clinical characteristics of all patients are reported in Table 1.

A diagnosis of diabetes mellitus was made according to the American Diabetes Association [17] before patient enrollment in the study. Most of the diabetic patients were already known by previous access to hospital. However, some of them were anamnestically known by relatives and supported by further analysis. Most of the diabetic patients were given insulin (81% and 87% in the TIA and AIS groups, respectively), alone or in association with oral antidiabetic drugs (59% and 51% in the TIA and AIS groups, respectively). Acute ischemic stroke (AIS) was defined as an episode of acute neurological dysfunction caused by focal cerebral ischemia, based on objective imaging techniques, such as a computed tomography (CT) or magnetic resonance imaging (MRI) scan, and clinical evidence of cerebral focal ischemic injury based on symptoms longer than 24 h. TIA was defined as a transient episode of neurological dysfunction (that disappeared within 24 h), caused by focal brain, spinal cord, or retinal ischemia, without acute infarction [18], according to the recent updated report from the American Heart Association/American Stroke Association [19].

### 4.1. Measurements

#### 4.1.1. miRNA Isolation and Real-Time Reverse Transcription

Circulating miRNAs were isolated, quantified, and converted in cDNA as previously described, by using Syn-cel-miR-39 miScripitmiRNA Mimic 5 nM (Qiagen, Milan, Italy) as an external control [6]. miRNA-195-5p, miRNA-451a, and Syn-cel-miR-39 expression levels were detected via qRT-PCR and analyzed by using the 2^^-ΔCT^ method of relative quantization [6]. Expression fold change data are reported as fold regulation values [20].

#### 4.1.2. Serum BDNF and VEGF-A Enzyme-Linked Immunosorbent (ELISA) Assays 

Serum BDNF and VEGF-A levels were measured using the Human BDNF ELISA Kit (ab99978, abcam, Milan, Italy) and the VEGF-A Human ELISA Kit (BMS277-2, Thermo Fisher Scientific, Milan, Italy), respectively, according to the manufacturer’s protocols.

### 4.2. Outcomes

Outcomes included plasma changes in both the miRNA-195-5p and miRNA-451a profiles, in diabetic vs. non-diabetic subjects with AIS or TIA upon admission (T0) and after 72 h of the post-ischemic period, as well as changes in the serum level of BDNF and VEGF-A in both AIS and TIA at admission (T0) and at 72 h during the post-ischemic period.

### 4.3. Statistical Analysis

Statistical analysis was conducted using repeated-measure analysis of variance (ANOVA), followed by Dunnett’s post-hoc test (as appropriate). Three independent experiments were performed for both the qRT-PCR and ELISA determinations. The correlation between BDNF or VEGF-A and miRNA levels was evaluated using Pearson correlation analysis. A probability of *p* < 0.05 was considered significant for all results, sufficient to reject the null hypothesis. The criterion of the differential expression of miRNAs was *p* < 0.05.

## 5. Conclusions

Herein, we reported that diabetic patients with stroke showed a two-fold larger expression of miRNA-195-5p and miRNA-451a in comparison to non-diabetic patients. This pattern was associated with lower BDNF and VEGF-A levels, suggesting a biochemical pattern underlying brain dysfunction and vascular damage. In non-diabetic patients with TIA, we also observed a complete decay of miRNA-195-5p and miRNA-451a expression 72 h after a post-ischemic stroke, paralleled by an increase in BDNF and VEGF-A levels, suggesting an attempt to preserve brain function and vascular integrity. Finally, miRNA-195-5p and miRNA-451a expression was inversely correlated with both BDNF and VEGF-A serum levels.

Since several already known biomarkers are not specific to ischemic stroke [16], novel and specific biomarkers are needed to better understand the mechanism involved in the pathogenetic chain of cerebral ischemia and the determinants of its severity.

Among the novel biomarkers for ischemic stroke (IS), circulating miRNAs look promising, since they better reflect specific brain pathophysiological alterations compared to the traditional IS biomarkers [21]. In this regard, the characteristic modulation shown by circulating miRNA-195-5p and miR-451a during the development of acute or transient brain damage, and their specific regulation in blood flow restoration mechanisms, may potentially lead to new strategies aimed at better understanding of pathogenesis of cerebral ischemia and an early prediction of stroke severity.

These data may suggest a role for these miRNAs as acute biomarkers in the pathogenesis and prognosis of diabetic AIS and TIA patients, but future studies are needed to evaluate a possible correlation between miRNA expression and severity of stroke.

## Figures and Tables

**Table 1:** Clinical characteristics in the control subjects (C) and in the diabetic (D) and non-diabetic (ND) patients with acute ischemic stroke (AIS) and transient ischemic attack (TIA). *N*, number of patients; M, number of male patients; HbA1C, glycated hemoglobin; BMI, body mass index; SBP, systolic blood pressure; DBP, diastolic blood pressure. The values are indicated as mean ± standard error of the mean (S.E.M.).

Characteristic	Control (C)	AIS		TIA		*p*-Value
		ND	D	ND	D	
*N* (M)	20 (10)	20 (10)	21 (10)	18 (9)	19 (8)	
Age (years)	71.4 ± 3	73.1 ± 3	70.4 ± 4	69.2 ± 3	72.1 ± 3	0.27
Diabetes duration (years)			17 ± 3		18 ± 5	0.78
HbA1C (%)			6.9 ± 0.6		7.1 ± 0.8	
BMI (kg/m^2^)	26.6 ± 3	25.4 ± 3	27.1 ± 4	25.2 ± 3	26.9 ± 3	0.71
SBP (mmHg)	139 ± 6	143 ± 5	145 ± 6	144 ± 5	145 ± 6	0.37
DBP (mmHg)	82 ± 3	84 ± 3	82 ± 4	83 ± 3	81 ± 3	0.64
Hypertension (%)	10 (50)	13 (65)	13 (62)	13 (72)	14 (74)	0.08
Smoking (%)	5 (25)	6 (30)	5 (24)	6 (33)	5 (26)	0.1
Hyperlipidemia (%)	8 (40)	10 (50)	10 (48)	8 (44)	9 (47)	0.1
